# Ethical issues in using ambient intelligence in health-care settings

**DOI:** 10.1016/S2589-7500(20)30275-2

**Published:** 2020-12-21

**Authors:** Nicole Martinez-Martin, Zelun Luo, Amit Kaushal, Ehsan Adeli, Albert Haque, Sara S Kelly, Sarah Wieten, Mildred K Cho, David Magnus, Li Fei-Fei, Kevin Schulman, Arnold Milstein

**Affiliations:** Center for Biomedical Ethics, University, Stanford University, Stanford, CA, USA; Department of Computer Science, Stanford University, Stanford, CA, USA; Department of Bioengineering, Stanford University, Stanford, CA, USA; Department of Psychiatry and Behavioral Sciences, University, Stanford University, Stanford, CA, USA; Department of Computer Science, Stanford University, Stanford, CA, USA; Department of Computer Science, Stanford University, Stanford, CA, USA; Clinical Excellence Research Center, Department of Medicine, Stanford University, Stanford, CA, USA; Center for Biomedical Ethics, University, Stanford University, Stanford, CA, USA; Center for Biomedical Ethics, University, Stanford University, Stanford, CA, USA; Center for Biomedical Ethics, University, Stanford University, Stanford, CA, USA; Stanford Institute for Human-Centered Artificial Intelligence, Stanford University, Stanford, CA, USA; Clinical Excellence Research Center, Department of Medicine, Stanford University, Stanford, CA, USA; Clinical Excellence Research Center, Department of Medicine, Stanford University, Stanford, CA, USA

## Abstract

Ambient intelligence is increasingly finding applications in health-care settings, such as helping to ensure clinician and patient safety by monitoring staff compliance with clinical best practices or relieving staff of burdensome documentation tasks. Ambient intelligence involves using contactless sensors and contact-based wearable devices embedded in health-care settings to collect data (eg, imaging data of physical spaces, audio data, or body temperature), coupled with machine learning algorithms to efficiently and effectively interpret these data. Despite the promise of ambient intelligence to improve quality of care, the continuous collection of large amounts of sensor data in health-care settings presents ethical challenges, particularly in terms of privacy, data management, bias and fairness, and informed consent. Navigating these ethical issues is crucial not only for the success of individual uses, but for acceptance of the field as a whole.

## Introduction

Concurrent advances in multi-modal sensing technology, machine learning, and computer vision have enabled the development of ambient intelligence—the ability to continuously and unobtrusively monitor and understand actions in physical environments. Ambient intelligence is increasingly finding use in health-care settings.^1,2^ Ambient intelligence involves using contactless sensors and contact-based wearable devices embedded in health-care settings to collect data (eg, images of physical spaces, audio, or body temperature), coupled with machine learning algorithms that efficiently and effectively interpret these data (figure). Applied to health-care settings, this technology can not only monitor a patient’s health status and trajectory, but also highlight the quality and nature of care delivered by the entire health-care team.

Ambient sensors are placed in hospital settings (eg, intensive care units [ICUs] and operating rooms, to monitor the activities of clinicians, staff, and patients, as well as in daily living spaces like independent living or community care settings, to gather data relevant to managing care for older people, chronic disease management, or mental health problems. In the hospital setting, ambient intelligence has been used to ensure the safety of clinicians and patients by monitoring the skill of a surgeon or adherence to hand hygiene protocols in the ICU.^3–6^ Outside of the hospital, ambient sensors in patients’ living spaces are able to monitor the status of frail older people or track a patient’s clinical trajectory for acute conditions.^7^ Facial recognition and voice data collected by sensors can be used to detect pain or validate identity for security purposes.^8,9^ By recognising and logging observed actions, ambient sensors in health-care settings can also help relieve staff of burdensome documentation tasks, which have been associated with clinical burnout.

For all its promise, ambient intelligence in health-care settings comes with a spectrum of ethical concerns that set it apart from other machine learning applications in health care. The continuous collection and storage of large amounts of sensing data involving various participants in different contexts, and the potential combination of many different types of data for analysis, raises issues of privacy, data protection, informed consent, and fairness that might not be easily addressed through existing ethical and regulatory frameworks.^10–12^ Health-care uses of ambient intelligence span clinical care, research, quality improvement, quality measurement, education and health-care employment. Stakeholders might include health-care professionals, health-care facility visitors, in-home family and non-family caregivers, and patients. Moreover, ongoing surveillance in health-care settings has social implications, including the potential for misuse and an effect on the clinical relationship. It is particularly important to formulate processes, and engage relevant stakeholders and expertise, for anticipating and addressing these challenges during the design and development of ambient intelligence applications. Thoughtfully navigating these issues is crucial, not only for the success of individual uses, but also for acceptance of the field as a whole. We review the issues raised by the use of this technology in various health-care domains, and discuss relevant ethical and legal constructs to help contextualise and respond to these issues.

## Developing ambient intelligence algorithms

To identify potential privacy and ethical issues that arise with ambient intelligence in health-care settings, it is first important to understand how these algorithms are developed. Learning-based ambient intelligence methods use data acquired from various ambient sensors and then apply machine learning and computer vision algorithms to identify specified patterns (including human behaviours in the videos) or to recognise speech in the audio.^13^ The table shows the stages for designing and implementing such algorithms. Decisions made at the start of the project about how to frame the research problem and to set forth desired outcomes provide the foundation for achieving relevant goals and avoiding problematic bias (stage 1). After data are collected and preprocessed (table, stage 2), the algorithms go through three major phases of model building: training, testing, and deployment (table, stages 3–5). In the training phase (stage 3), the algorithms are given examples of images, videos, or other sensory data often with associated so-called ground truth labels that annotate objects or actions of interest. An algorithm to detect when a patient gets out of bed might be fed hours of videos, each annotated with timestamps (ie, sequences of characters or encoded information) that denote the start and end of when patients are getting out of bed. These labels or annotations are often done by hand, with a person (or many people) reviewing raw data and adding the necessary labels.

Just as a student who has seen the test questions before an examination might get an artificially high score, algorithms tend to do unexpectedly well if their performance is evaluated using the same data that was used to train them. To ensure trained models can generalise to unseen data, researchers use a separate labelled validation dataset during the training stage. The validation dataset is like online practice exams; it is repeatedly used to evaluate and tune the algorithm during the training process. Once the algorithm has achieved a satisfactory score on the validation dataset, it is evaluated against the test dataset (stage 4); this is like the final exam, in which the algorithm is run against never-before-seen data, and its final performance characteristics are reported.

In most commonly used implementations of machine learning (ie, supervised machine learning), successful training, validation, and testing are only possible with labelled data, and in large amounts. Annotation is the process of labelling activities or behaviours of interest, and is a manual process in which a person has to look back at the data to determine if, when, and where an activity of interest is occurring in the data.^14^ The data required for this stage needs to be of sufficiently high quality to enable an observer to discern the activity of interest—eg, for classification of fine motor skill activities, high-resolution images are vital.

The next stage in the process is deployment in the target health-care setting (stage 5). In the deployed stage, the sensory data are generally subject to assessment only by the algorithm, which can be used to provide direct interventions for quality improvement or to assist clinicians in making decisions. Although active learning methods can be used to create machine learning algorithms that can receive feedback loops from the experts (ie, clinicians in health-care settings), such algorithms are rare in the application of ambient intelligence to health-care environments.^15^ At this stage, research questions can change to whether or how the algorithm affects clinical care and, ideally, patient outcomes. Research focus can also shift to quality improvements based on the application of the algorithm (stage 6). Algorithm performance can vary after moving from training data to target data, a phenomenon known as domain shift.^16^ For instance, an algorithm can fail if it sees an unfamiliar room layout that it never saw during the training process.^17^ Unexpected results like this need to be investigated, which often involves a person viewing the images to understand the mode of failure and design a solution.

A deployed algorithm is necessary but usually not sufficient to derive benefit from ambient intelligence—the output of that algorithm must be connected to some clinical workflow or action. Does the output of the algorithm automatically result in a decision or action, or is there a person in the loop who is shown a notification and must then decide on how to act, in which case is it better to err on the side of alerting too much or too little? What are the clinical or operational metrics that measure success? These questions form the bridge between a technically high-performing algorithm and an actual benefit to patients or other stakeholders. The deployment stage raises questions regarding how to test the algorithms in the clinical environment. In the USA, if the sensors are integrated with the algorithms, they might be classified as medical devices, and thus subject to regulation by the US Food and Drug Administration.

As our understanding of how to develop this technology improves, the list of actions or behaviours of interest to the research community might also grow. Previously annotated images can be reannotated to discern an additional set of activities for labelling (or researchers might be interested in a finer gradation of previously labelled activities). Furthermore, building increasingly large databases of labelled images could improve the performance of algorithms over time.

The development of ambient intelligence also requires engagement with various ethical issues at each stage of the research process. Broad ethical frameworks for artificial intelligence and machine learning usage already exist. It will be important to go beyond lists of broader principles to develop tools and processes for ambient intelligence usage that incorporate active and ongoing reflection and engagement with ethical issues in the design and development of these applications—for example, identifying the stages of development at which to engage stakeholders’ perspectives or incorporate ethical consultation. Ethical issues in the stages of ambient intelligence development and use in health-care settings are summarised in the table, and described in further detail in the following sections.

## Ethical issues

### Privacy

Researchers developing ambient intelligence applications need to carefully consider various aspects of the project, including the settings in which sensing data will be collected, the types of information that could be captured by the sensors, the inferences that might be drawn from that information, and what design measures might be needed to protect that information, especially given that efforts to deidentify information cannot be as complete as is sometimes imagined. In the USA, privacy interests are protected under constitutional law, a variety of federal and state statutes and regulations, and by cultural norms and professional ethics.^18^ Engagement with privacy concerns for ambient intelligence usage should aim to go beyond basic compliance with relevant laws and address the different values and trade-offs involved in privacy interests. The context of the specific use of ambient intelligence will influence which ethical framework is used to evaluate these trade-offs and interests. Different ethical and regulatory frameworks apply to different types of health-care activities and stakeholders, which should be taken into account when developing an ambient intelligence application. Doing a project as research rather than as quality improvement, for example, determines which legal requirements will apply regarding privacy and informed consent.

Privacy is a concept that incorporates a range of rights and obligations meant to protect an individual from unwanted intrusions or interferences into their personal domain.^19^ In artificial intelligence projects and health-care settings, there is often a focus on informational privacy, which involves “how and when personal information should be communicated or obtained and what uses of it will be made by others, and encompasses the collection, storage, use, maintenance, dissemination/disclosure, and disposition of personal information”^.20,21^ Ambient sensors will potentially collect data on various people in health-care settings, which can include patients, doctors, postgraduate trainees or residents, nurses, hospital staff, family and friends of the patients, and others. Depending on the specifics of the hardware, an ambient sensor can capture a range of attributes of a person, including a person’s face, voice, heart rate, gait, and parts of their bodies, or data that can reveal IP addresses. Data like these could lead to the personal identification of a person or to public exposure of personal information regarding their health status or activities.^22^ Data collected through ambient sensors could also be used to make predictions regarding health outcomes, particularly if different types of data (eg, body temperature, respiration, and voice) are collected and analysed together.^23^ Participants might not be aware of how their data can be analysed for predictive purposes or the additional health inferences that could be drawn from their data.^24^

Informational privacy is not the only type of privacy concern that could be an issue. Ambient sensors could be placed in patients’ homes or many health-care settings that patients and hospital staff, caregivers, family, and others might ordinarily expect to be free of monitoring devices. Some people might want to restrict when a third party is able to view particular parts of their bodies or monitor them in a vulnerable state, such as when they are going to the bathroom. The rights of an individual to make decisions about their own care and activity, without undue interference from government or unauthorised people, is a different aspect of privacy, sometimes referred to as decisional privacy.^25^ Protections for decisional privacy involve the timing and type of consent required, and who needs to give consent. The stage of data collection and algorithmic development are relevant to some of these privacy concerns. For example, it might be relevant for stakeholders to know who has access to the data at different stages of project development, and whether an algorithm or a person is seeing the data.

Privacy is a value that presents trade-offs with other values and considerations in a project. The type of project (eg, research versus quality improvement) is relevant to the ethical framework used to assess such trade-offs. For example, using thermal imaging instead of full video capture can obscure the identity of participants, but this must be weighed against other goals, such as whether thermal imaging can adequately capture the features relevant to the scientific goals of a project. Privacy provisions in medical research generally balance individual privacy protection with the need to promote data sharing for scientific purposes. It is important that ambient intelligence researchers collecting data from ambient sensors are able to clearly articulate the benefits to be derived from the research, to facilitate the assessment of how those benefits balance against risks to privacy and to formulate measures to preserve participants’ privacy accordingly.

It should not simply be assumed that patients or other participants value informational privacy over other types of privacy or the potential scientific benefits from allowing the collection of some of their personal information. There are indications that people might be willing to share personal information if they feel it is for the benefit of science.^26^ Furthermore, people might feel that some types of sensing data raise more privacy concerns than others. For example, people might be more concerned about the recording of conversations than of imaging data. It is important to engage stakeholder perspectives during the development of ambient intelligence projects to help formulate appropriate ways to balance privacy protections against other interests.

Choices regarding the context of the project, and the types of stakeholder involved, effect which laws and regulations will be relevant for preserving privacy. The Health Insurance Portability and Accountability Act (HIPAA) Privacy Rule generally applies to projects sponsored by health-care organisations in the USA. For projects classified as human subjects research, privacy and data protection measures are required as part of the ethical conduct of research. If the activities of medical or other health-care students’ activities are captured by the sensors, privacy protections under the Family Educational Rights and Privacy Act of 1974 might also be applicable to their participation. The applicability of state and local data privacy or biometric statutes should be ascertained for a project. For example, the California Consumer Privacy Act provides for consumer rights in relation to the access, sharing, and deletion of personal information collected by businesses, which can apply to some health-care settings and data.^27^ The EU Data Privacy Regulation (GDPR) is broader than HIPAA, and defines personal data as information connected to an identified or identifiable person.^28^ Thus the GDPR can apply to information like video images and IP addresses. Furthermore, US health systems can be liable under the GDPR if they have European patients.^29^

The HIPAA Privacy Rule requires informed consent, or a waiver of authorisation or documentation of informed consent, to use protected health information for specific research studies,^30^ but there are no restrictions on the use or disclosure of deidentified information.^31^ HIPAA sets out two different approaches to deidentifying patient data: safe harbour and expert determination. The safe harbour method requires removal of all of 18 specified personal identifiers, which includes names and dates, as well as biometric identifiers, such as finger and voice prints and full-face photographs and any comparable images.^32^ Thus, such deidentification of visual sensor data in clinical settings might not be possible. State laws or local institutional review boards might require additional deidentification measures. The expert determination approach requires an expert to assess that “the risk is very small that the information could be used, alone or in combination with other reasonably available information, by an anticipated recipient to identify an individual who is a subject of the information”.^33^ Using this standard, careful restrictions on access to the data and an inability to combine data could achieve the deidentification standard. For example, consider an annotator working at a secure terminal that prevents copying image data and prevents combining data (eg, image data with internet search data). In such a case, the data could be considered deidentified using this standard. Although either approach to deidentification might be appropriate for a specific ambient intelligence project, the expert determination approach is likely to be more expensive, but is easier to tailor to the project at hand and more consistent with providing accurate assurances regarding the completeness of deidentification on offer.

It is important to note that the risk of reidentification of data cannot be completely eliminated.^34,35^ Personal information is increasingly available in large online databases, and trends towards data aggregation and data analytics make it more likely that data can be reidentified. Studies have shown that a range of different types of health data that were deidentified by traditional means can be reidentified.^36,37^ At best, approaches can be taken to minimise the risk of reidentification.^38^ At the same time, developing deidentification approaches could be important in ambient intelligence research, such as developing scalable approaches to video annotation by using outside contractors to do specific tasks during development. Rather than having deidentification as a standard, analysis of protected health information in accordance with HIPAA requires comprehensive data privacy and security standards, and training for all individuals who have access to the data.

### Data management and liability

A key tenet of privacy in research on humans is stewardship of the data. Effective stewardship includes ensuring that only members of the research team have access to the study data, that members of the research team are trained in the areas of data privacy and security and have signed privacy agreements with the sponsoring institutions, and that data practices include minimising access to fully identifiable data as much as possible (eg, by substituting study identification numbers for identifiable names). Research in ambient intelligence, as projects continuously collect data, could also contribute to creating standards for data sharing and data merging, such as methods to collect data not only for ongoing processes, but also for the contexts of those processes.^39^

The HIPAA Privacy Rule requires covered entities to consider issues such as the technical, hardware, and software infrastructure of their data security measures, to protect health information. Privacy considerations include the careful assessment of security measures, including data storage and transfer. Data for computer vision and other sensor data constitutes a large amount of information to be stored for research purposes. In such cases, technical issues that drive the research (eg, compression, frame rate capture) could also increase or reduce these requirements. Data encryption is a crucial element of protecting patient privacy. New technology (eg, edge computing) can allow encryption before data is transmitted from the computer vision camera to the data storage destination (eg, local server or protected cloud environment). Given the storage requirements, careful consideration is required about how long the raw data will be maintained. At the research stage, this length of time will be driven by the research requirements. In the production stage, institutional data retention policies based on local law might need to be developed (given the scale of raw data being collected, data retention might be challenging if patients have multiple video sensors operating continuously).

During the data annotation stage, data is sometimes sent to an outside business for annotation. HIPAA includes provisions for the sharing of protected health information with business associates. Still, it remains essential for a project to carefully consider the data security practices of the company providing data annotation services.

The raw imaging data collected by sensors could be relevant to potential legal actions to establish liability.^40^ For example, if a patient is injured or suffers an adverse event, there could be a request to see the sensor data to determine who or what might have caused the event. Sensors could also capture illegal activities, such as the use of illicit substances or abusive behaviour. In the data annotation and testing phases of the research, readers might observe these events. In the production phase, the computer would not detect these events because the algorithms will only recognise behaviours on which they are trained. Ambient intelligence projects thus need to consider carefully (depending on what type of analysis of the sensor data is done) if, when, and how any problematic behaviours discovered in a research setting would need to be reported, and to whom. Because data annotation might not occur until long after the data are recorded, or might never occur for an image that is collected, a research project cannot provide assurances to patients or participants regarding the detection of these behaviours. A US National Institutes of Health certificate of confidentiality can be used to prevent the discovery of raw image data during legal proceedings.^41^ However, if a project is proceeding as a quality improvement study, it is not considered to be research, and might not be eligible to receive a certificate of confidentiality.

### Consent

Participants in research studies that collect data through ambient intelligence have the same rights and concerns as patients in other types of research on humans. In considering whether to participate in the project, people would need to be aware of the potential use of their data, including how their data might be used for the specific research project underway, future research efforts, and potential collaborations with other investigators (at other institutions or within industry). A description of the ambient intelligence research project needs to address potential expectations regarding the data, such as letting the patient and their family members know that the sensor data cannot be expected to provide warnings of real-time patient problems, because a substantial amount of time might pass before the capture of sensor data and its review (not all sensor data might be needed for annotation, so there should be no expectation that specific data will be reviewed). Patients should be aware that their care will not be affected by their participation in the study (unless that is the purpose of the study) or by their withdrawal from the study. Also, patients should understand that their care team is not their research team, and that data annotation will not be done by the care team.

A waiver of informed consent is permitted if the institutional review board determines that the research involves minimal risk to participants, the research cannot be practically carried out without a waiver, the waiver will not affect the rights or welfare of the participants, and (if appropriate) the participant will receive additional information regarding their participation.^42^ Many ambient intelligence projects could probably be classified as minimal risk, if most of the risk relates to patient or participant privacy. In contrast, other settings or project designs might involve more access to health data or larger privacy repercussions, and thus be of such sufficient risk to a participant that full consent would be necessary. Some hospitals or other health-care institutions might already include notice of, or consent for, research on the forms or documents given to patients. Therefore, ambient intelligence projects might need to consider other consent measures that are applicable at the institution.

Documentation that a project meets the requirements for a waiver of consent could be useful in settings such as an ICU or emergency department. Institutions might want to ensure patients are made aware of ambient intelligence via notices of privacy practices in their patient consent forms. For example, a hospital consent form that notifies patients about the use of their medical data might not be sufficient to constitute consent for research purposes for this type of project, so an additional consent process would be needed. Even when there are not applicable legal requirements for informed consent, it is important to provide transparency regarding the use of ambient intelligence systems in particular settings to maintain public trust and provide people with the opportunity to make decisions regarding their personal information. If facial recognition technology is used, the Association for Computing Machinery recommends providing ongoing public notice at the point of use in a format appropriate to the context.^43^ Furthermore, if sensors are collecting audio data, state law regarding consent for audio recordings is likely to be applicable.

### Fairness and bias

The potential for bias in artificial intelligence systems is a recognised challenge for the implementation of artificial intelligence systems in healthcare.^44–46^ Machine learning processes for artificial intelligence generally use a massive set of data input to produce the desired output by finding patterns in the data.^47^ Using large amounts of data, the artificial intelligence model is trained to identify patterns and create rules that adjust and improve the model’s parameters.^48,49^ Although various methods have been developed to mitigate bias during model training, there are still several ways in which machine learning systems can unintentionally produce bias.^50,51^ First, the accuracy of the machine learning algorithm depends on the quality of the training and validation datasets.^52^ For example, one algorithm might choose thermal, colour video, or depth imaging for the ambient intelligence project. If the dataset does not reflect the relevant qualities of the population to which the algorithm is applied, then there can be bias in the outcomes. The absence of sufficient geographical distribution of patient cohorts used to train algorithms is another potential source of systemic bias.^53^

Before artificial intelligence, medical datasets and trials had a long history of bias and inadequate representation of women and people of different races and ethnicities.^54^ If the dataset used for machine learning is not sufficiently inclusive of people of different sexes, races and ethnicities, or socioeconomic backgrounds, this is one way in which the results can be biased.^55^ COVID-19’s disparate effect on some racial and ethnic groups reflects long-standing racial disparities in medical research and access,^56^ and is a stark reminder of the need to mitigate bias in emerging technologies for health research. Depending on the specific project, the sensing data might need to be classified in terms of which behaviours or activities are deemed normal and which are not, to decide what information will lead to specific interventions in the health-care setting, once the ambient intelligence is deployed. Because of the role of annotators in labelling data, appropriate diversity among annotators might need to be considered for a given project. Sensitivity to cultural and other types of differences in behaviours and activities monitored by the ambient sensors is important in these determinations.

Bias can also result if algorithms used for a particular purpose or context are transferred to a new context, for example, if an algorithm used in an urban context is transferred to a rural context.^57^ Furthermore, machine learning bias can stem from an absence of alignment between the type of information used to develop an algorithm and the expectations of the user. This mismatch can lead to misinterpretation of the algorithm, which produces a bias in the outcomes for patients.^58^ For research projects that involve imaging of specific patient populations, it is important to consider the generalisability of findings drawn from that particular population. Inclusion and exclusion criteria for clinical studies can also introduce bias.^59^ Efforts to recruit and obtain the consent of people from diverse populations for the training dataset will need to engage with the different perspectives and contexts of various groups regarding protection of personal data and consent. These efforts should include consideration of how the research might benefit and burden different populations. The Association of Computing Machinery provides relevant recommendations, such as ensuring that a system’s initial and dynamic biases and inaccuracies are understood before being used to support decisions that affect individuals’ human and legal rights, and providing for systems to be auditable by third-party monitoring.^60^ The Association states that when error rates are reported, the context for those errors should be identified and addressed in standards for those error rates, and reported error rates should be disaggregated by sex, race, and other context-dependent demographic features where relevant.61 Beyond these principles, there remains a need for ambient intelligence research to support diversity among researchers working on projects, including the people doing the annotation.

## Social implications

As with other artificial intelligence technologies introduced into health care, ambient intelligence is expected to have an effect on the clinical relationship.^43^ The use of ambient intelligence systems takes the practice of medicine further from the traditional dyadic physician—patient relationship. For example, for some uses, the ambient intelligence itself could be viewed as a third party to the health-care encounter, whereas with other uses, the ambient intelligence might be part of a system in which clinical decision making is guided heavily by default rules set by the health-care organisation.^62^ Another concern is about how patient trust in the clinical relationship would be affected by normalised, ongoing health-care surveillance. Ambient intelligence in the workplace is also likely to effect the employer—employee relationship, in turn affecting perceptions of responsibilities, trust, and obligations.^63^ Continuous monitoring by ambient intelligence could relieve some of the burden on human caregivers, but also could affect how clinicians view their responsibilities within the clinical relationship, such as the responsibility to identify areas of required change in the health-care setting. The use of ambient intelligence systems could potentially increase clinicians’ exposure to liability for their clinical judgments, and raises questions about the proper balance of liability between clinicians and software used for specific purposes.^61^

Ambient intelligence applications need to be scrutinised for potential unintended consequences. Some ambient intelligence uses will involve the creation of new software systems that allow for long-term surveillance of individuals and their activities, and analysis of massive amounts of sensor data. It is not a stretch of the imagination to think that this type of software would be of interest to other institutions or organisations, inside or outside health care, for a purpose that might be ethically problematic, such as various applications for tracking or identifying movements. Thus, ambient intelligence projects will need to consider whether the system or software will eventually be sold to other parties (this concern should include the algorithms themselves, any technology developed in the course of the research, as well as research methods, and whether any data are included in the transfer). Unfortunately, it is not clear how much control research teams will have over the unintended consequences of their research, especially if the work produced is a paper outlining approaches to the development of machine learning tools. However, there are increasing calls for systems developers and users to be accountable for the consequences of the use and misuse of computer systems.

Use of ambient intelligence raises concerns about the increasing use of surveillance technology throughout society. Ambient intelligence in health-care settings can serve to further normalise surveillance, while minimising considerations of the burdens placed on specific groups, or society as a whole, by such practices. Moreover, it should not simply be assumed that the collection of detailed and comprehensive data on patients and activities associated with physical health will produce a scientific benefit. Ambient intelligence projects need to be developed with careful consideration of how the burdens and benefits of the research will be distributed and experienced by various stakeholders. Potential uses of ambient intelligence in health-care settings should be evaluated according to whether successful implementation will mainly benefit people of higher socioeconomic status or specific demographic groups. Additionally, because one area of focus for ambient intelligence applications is in monitoring older people in hospitals and home health-care settings, it is important to develop technology and guidance that is specific to supporting the needs of this population.^64^

Engaging stakeholders, including at the beginning of a project’s development, is a key aspect of ethical implementation of ambient intelligence. Ahonen and colleagues^65^ argued that a formalised risk assessment process is necessary for many types of ambient intelligence projects, and a key component of their proposed approach is to give stakeholders the opportunity to participate in the process of assessing, and identifying solutions for, risks posed by ambient intelligence. Transparency and informed consent are core components of addressing concerns about surveillance and privacy. At the same time, research in related fields that involve continuous capture of a person’s digital data suggests that even when people are informed of, and consent to, continuous data collection, they eventually become less vigilant and forget to manage their behaviour accordingly, thus revealing information or activities they might not have meant to expose to surveillance.^66^ Thus, there could be a need, with ambient intelligence systems, to consider ways that participants might need reminders, through visual cues or otherwise, of the ongoing collection of sensor data.

## Conclusion

In this Viewpoint, we reviewed some of the ethical issues facing the research community during the development of ambient intelligence in health-care settings. Researchers pushing the frontiers of ambient intelligence uses for health care will need to anticipate and address these issues. For example, although privacy is a crucial concern, privacy considerations beyond data protection need to be balanced with other interests and values. Interdisciplinary collaboration will be valuable in identifying and addressing potential ethical issues. Engaging people who have relevant expertise outside of research teams, and the stakeholders themselves, brings useful perspectives to minimise potential harms to participants. Collaboration and engagement early in the process of the design of ambient intelligence systems are important to support the transparency and accountability necessary to make them trustworthy additions to the health-care system.

## Figures and Tables

**Figure:**
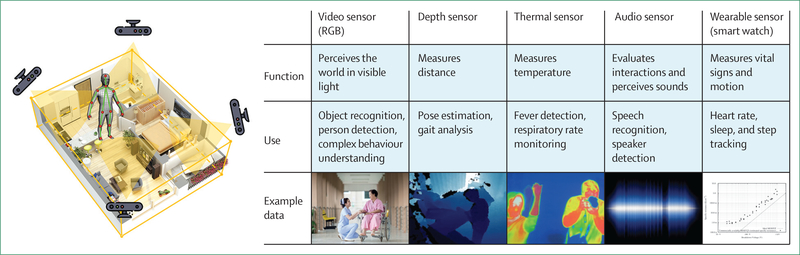
Sensor data collection for ambient intelligence in health-care settings RGB=red, green, blue analogue colour video signal.

**Table:** Stages of designing and implementing algorithms for ambient intelligence use in health-care settings

	Activities	Key points	Ethical issues

Stage 1: framing the problem	Decide what the statistical model should achieve	Articulate the desired outcome, which also shapes what data will be needed	Setting up a project to achieve relevant goals and avoid problematic bias
Stage 2: data collection	Inclusion and exclusion of data	Including relevant data and avoiding an approach that reinforces existing prejudice and biases in the context of the problem; includes the issue of primary use (whether data were generated or collected specifically for the algorithm) and secondary use (whether data from another source are being repurposed)	Avoiding problematic bias; privacy; consent
Stage 3: training and validating the algorithm	Annotation—ie, activities and behaviours are labelled	Quality requirements for the image or sensory data will be determined by the behaviour or action of interest	Privacy; fairness and bias
Stage 4: testing	Assess computer performance in applying a label to input data (eg, image or video)	Could require annotation to be done again by people	Privacy; liability
Stage 5: deployment	Validated algorithm deployed in the care setting	Image or other sensory data are assessed only by the algorithm, with no labelling being done by people	Privacy; achieving appropriate care decisions; avoiding misinterpretation and bias; liability
Stage 6: long-term use	Ambient intelligence system used to collect data	Continuous monitoring by the sensor is required; use of ambient intelligence affects health-care decision making	Fairness; privacy and surveillance; effect on clinical relationship; effect on health-care employer-employee relationships; potential for misuse
